# CD200 and Chronic Lymphocytic Leukemia: Biological and Clinical Relevance

**DOI:** 10.3389/fonc.2020.584427

**Published:** 2020-11-26

**Authors:** Giovanni D’Arena, Vincenzo De Feo, Giuseppe Pietrantuono, Elisa Seneca, Giovanna Mansueto, Oreste Villani, Francesco La Rocca, Fiorella D’Auria, Teodora Statuto, Luciana Valvano, Francesca Arruga, Silvia Deaglio, Dimitar G. Efremov, Alessandro Sgambato, Luca Laurenti

**Affiliations:** ^1^ Hematology, “S. Luca” Hospital, ASL Salerno, Vallo della Lucania, Italy; ^2^ Department of Pharmaceutical and Biomedical Sciences, University of Salerno, Salerno, Italy; ^3^ Hematology and Stem Cell Transplantation Unit, IRCCS Centro di Riferimento Oncologico della Basilicata, Rionero in Vulture, Italy; ^4^ Laboratory of Preclinical and Translational Diagnostics, IRCCS Centro di Riferimento Oncologico della Basilicata, Rionero in Vulture, Italy; ^5^ Laboratory of Clinical Research and Advanced Diagnostics, IRCCS Centro di Riferimento Oncologico della Basilicata, Rionero in Vulture, Italy; ^6^ Cancer Immunogenetics Unit, Department of Medical Sciences, Molecular Biotechnology Center, University of Turin, Turin, Italy; ^7^ Molecular Hematology, International Centre for Genetic Engineering and Biotechnology, Trieste, Italy; ^8^ Scientific Direction, IRCCS Centro di Riferimento Oncologico della Basilicata, Rionero in Vulture, Italy; ^9^ Hematology Institute, IRCCS Fondazione Policlinico Universitario A. Gemelli, Rome, Italy

**Keywords:** CD200, chronic lymphocytic leukemia, prognosis, diagnosis, flow cytometry

## Abstract

CD200, a transmembrane type Ia glycoprotein belonging to the immunoglobulin protein superfamily, is broadly expressed on a wide variety of cell types, such as B lymphocytes, a subset of T lymphocytes, dendritic cells, endothelial and neuronal cells. It delivers immunosuppressive signals through its receptor CD200R, which is expressed on monocytes/myeloid cells and T lymphocytes. Moreover, interaction of CD200 with CD200R has also been reported to play a role in the regulation of tumor immunity. Overexpression of CD200 has been reported in chronic lymphocytic leukemia (CLL) and hairy cell leukemia but not in mantle cell lymphoma, thus helping to better discriminate between these different B cell malignancies with different prognosis. In this review, we focus on the role of CD200 expression in the differential diagnosis of mature B-cell neoplasms and on the prognostic significance of CD200 expression in CLL, where conflicting results have been published so far. Of interest, increasing evidences indicate that anti-CD200 treatment might be therapeutically beneficial for treating CD200-expressing malignancies, such as CLL.

## Introduction

Mature B-cell leukemias are heterogeneous in clinical and biological features. Despite the large body of studies published, difficulties to get a firm diagnosis still exist in some cases due to the lack of disease-specific markers and overlapping immunophenotypes. CD200 has recently emerged as a useful tool to better discriminate among several chronic leukemias. In addition to the usefulness of this marker in the diagnostic setting, it also has a prognostic role and may represent a potential therapeutic target in chronic lymphocytic leukemia (CLL).

In this review, we provide a summary of published data on the biological and clinical relevance of CD200 in CLL, identified through a literature search of the MEDLINE, Google Scholar, and Scopus databases, aiming at providing an update of the published literature on this topic. The search comprised the terms “CD200”, “chronic lymphocytic leukemia”, and “chronic B cell leukemias” without a date restriction. All articles and Meeting Abstracts we found were evaluated and included in this review.

## CD200 Antigen and CD200 Receptor

The surface membrane glycoprotein CD200, formerly termed OX-2, is encoded by the 29,744 bp long CD200 (OX-2) gene located on the long arm of chromosome 3 (3q13.2) (1).Three transcript variants of CD200 are known: variant 1, 2,226 bp long, containing 7 exons and encoding the 269 amino acids long isoform a; variant 2, 2,301 bp long, containing 7 exons and encoding the 294 amino acids long isoform b; and variant 3, 2,085 bp long, missing an exon and encoding the 153 amino acids long isoform c (2).

**Figure 1:**
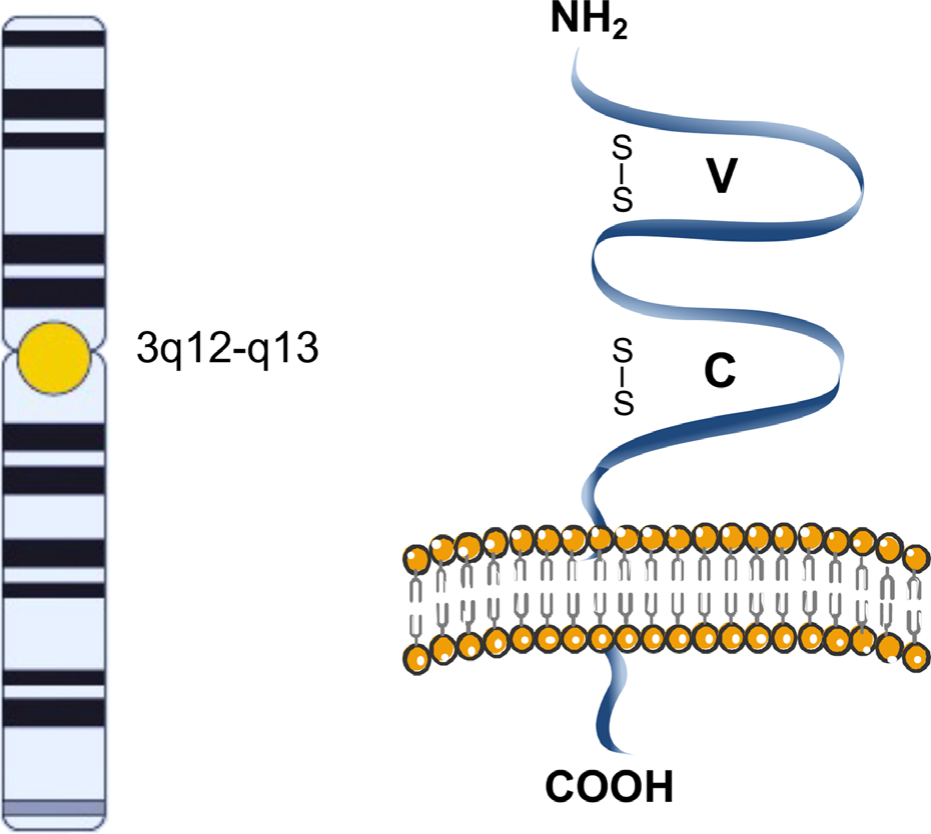
Schematic representation of CD200. The CD200 glycoprotein has two extracellular like Immunoglobulin domains formed by disulfide bonds, one variable (V) and one constant (C), a single transmembrane region, and a cytoplasmic tail.

The CD200 glycoprotein is a single-pass, type I, highly conserved membrane protein, belonging to the immunoglobulin superfamily, spanning the membrane once, with the N-terminus on the extracellular side of the membrane (2). It is composed of two extracellular (one variable and one constant) immunoglobulin-like domains, a single transmembrane region, and a cytoplasmic tail (**Figure 1**) (3).

The receptor for CD200 (CD200R) also has two immunoglobulin-like domain. The phylogenetic analysis demonstrated that the receptor is closely related to CD200 and probably evolved by a gene duplication (2). As a matter of the fact, the genes for human CD200 and CD200R are closely linked on chromosome 3 (4). However, the CD200R has a longer cytoplasmic tail with signaling motifs different from CD200 (5).

CD200 is normally expressed on a variety of cell types, including thymocytes, B lymphocytes, a subset of T lymphocytes, neurons, endothelial cells, some dendritic cells, kidney glomeruli, and syncytiothrophoblasts (6). The expression of CD200R is more restricted and includes myeloid leucocytes, such as macrophages, dendritic cells, and mast-cells, as well as B lymphocytes and a subset of T lymphocytes (7, 8).

## Function of CD200 and Its Role in Cancer

CD200, by means of the interaction with its receptor, induces the suppression of T-cell mediated responses, limiting inflammation in a wide range of inflammatory diseases (**Table 1**). The inhibition of macrophage function, induction of regulatory T-helper cell type (Th1) to Th2 cytokine profile switch, suppression of natural killer cell function, and inhibition of tumor-specific T-cell immunity have been all experimentally demonstrated (9–14, 15–18). Consistent with its immunosuppressive role, CD200-deficient mice are susceptible to tissue-specific autoimmunity (15). Gorczynski and co-workers demonstrated that the interaction of CD200 with CD200R is able to decrease the production of Th1-like cytokines, such as interleukin (IL)-2 and interferon (IF)-γ, and increase the release of Th2-like cytokines, such as IL-10 and IL-4 (16). In addition, the same group reported that the CD200/CD200R interaction induces the *in vitro* differentiation of T lymphocytes toward CD4^+^CD25^+^Foxp3^+^ regulatory T-cells (Tregs) (16).

**Table 1:** CD200:CD200R interaction and negative control of immunity.

KEY FACTS
Reduced Th1 cytokine (IL-2, IFNγ) production (9)
Increased IL-10 and IL-4 production (9)
Induction of Tregs (10)
Inhibition of mast cell degranulation (11, 12)
Downregulation of basophilic function (13)
Suppression of natural killer cell function (14)

CD200R, receptor of CD200 antigen; Th, T helper; IL, interleukin; IFN, interferon; Tregs, regulatory T-cells.

Moreaux et al demonstrated that CD200 mRNA is overexpressed on cells of several types of cancers compared to their normal counterparts, including chronic lymphocytic leukemia (CLL) (19). Kretz-Rommel et al elaborated a tumor model on the basis of the previous demonstration that CD200 is up-regulated in CLL and that the up-regulation in multiple myeloma (MM) and acute myeloid leukemia (AML) correlates with adverse prognosis (20–22). These authors firstly demonstrated that human peripheral blood mononuclear cells (hPBMCs) and Namalwa tumor cells (Burkitt’s lymphoma cell line lacking CD200 expression) simultaneously injected in NOD/SCID mice show reduced tumor growth with respect to that observed in mice in the absence of hPBMCs (23, 24). When the Namalwa tumor cells were engineered to express human CD200 on their surface and simultaneously injected with hPBMCs, CD200 expressed on tumor cells prevented hPBMCs from eradicating the cancer cells. This tumor model clearly demonstrated the immunosuppressive activity of CD200. Moreover, treatment with anti-CD200 monoclonal antibodies (Abs) was also highly effective in this model and inhibited the growth of Namalwa CD200 tumor cells in NOD/SCID hu-mice by >90%. The same study showed that CD200 is a marker of activated T cells and that the use of IgG1 anti-CD200 (a constant region variant that can mediate ADCC) resulted in efficient target cell killing of these lymphocytes by ADCC. Taken together, these data have relevant implications for the immunotherapy of cancer patients with anti-CD200.

## Relevance of CD200 in Diagnosis of Chronic Lymphoproliferative Disorders

The neoplasms of mature B cells are heterogeneous diseases currently included in the “mature B-cell lymphoid neoplasms” of the WHO classification (25, 26). Immunophenotypic profile, cytogenetics, and molecular biology must be all taken into account as a multidisciplinary integrated approach to differentiate the disease entities belonging to this category. However, difficulties in defining some cases still exist. Diagnostic accuracy is crucial in diagnosing neoplasms that require different specific treatments.

Flow cytometric characterization of chronic lymphoproliferative disorders represents a cornerstone in the diagnostic approach to neoplasms of mature lymphocytes (27). In the early 90s, a British group in London proposed a scoring system (Matutes score), which was based on analysis of 5 membrane markers: CD5, CD22, CD23, FMC7, and surface immunoglobulin (SmIg) (28). A score of 1 was assigned for each of the following immunophenotypic features: CD5 positive, CD22 weak or negative, CD23 positive, FMC7 negative, SmIg weak. The total score, according to this scoring system, is usually 4 or 5 for typical CLL cases, and 3 or less for other mature B-cell lymphoid neoplasms (28). A few years later, the same group improved the diagnostic accuracy of the score (from 91.8% to 96.8%) simply replacing CD22 with CD79b (29).

**Figure 2:**
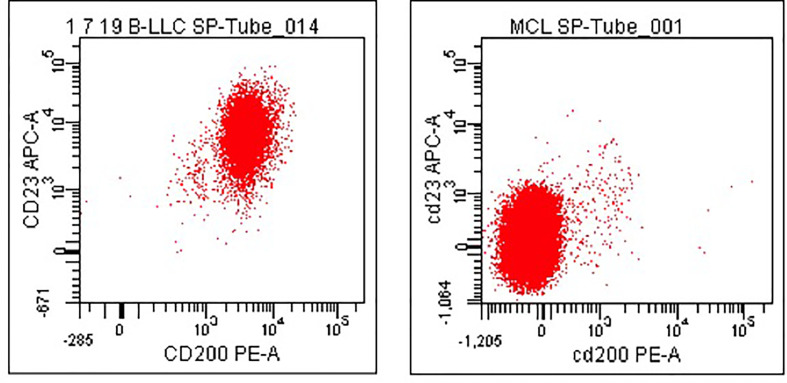
CD200 expression in CLL and MCL. Dot plots showing a case of CLL in which both surface CD200 and CD23 were expressed (left panel) and a case of MCL in which both antigens were found negative (right panel). B-cells only have been gated for the analysis.

The Matutes score is used worldwide as a diagnostic tool. However, some cases of chronic lymphoid neoplasms are still misdiagnosed, including some cases belonging to a more aggressive entity, such as mantle cell lymphoma (MCL). In this context, CD200 has been shown to have differential expression in B-cell neoplasms and to well discriminate CLL from MCL and hairy cell leukemia (HCL) and its variant form (v-HCL) (**Figure 2**) (30–32).

The reports on the relevance of CD200 expression to differentiate B cell chronic lymphoid neoplasms that have been published so far are listed in **Table 2**. Firstly, Palumbo et al in 2009 demonstrated that CLL can be differentiated from MCL according to the expression of CD200 (all patients with CLL expressed CD200, whereas all patients with MCL were negative) (31). At the same time Brunetti et al, analyzing only patients with HCL, showed that all patients with typical HCL expressed CD200 (30). Shortly after, Dorfman & Shahsafaei studied by means of flow cytometry bone marrow and lymph node aspirates from patients with different mature B cell lymphoid neoplasms, showing that CLL and HCL cases were all CD200 positive, prolymphocytic leukemia (PLL) were positive in 80% of cases, while MCL, splenic marginal zone lymphoma (SMZL), and follicular lymphoma (FL) were all negative (32). Since then, several other researchers analyzed samples from patients with mature B-cell lymphoid neoplasms (33–63). Overall, the published data confirmed the positivity in all patients with CLL and HCL and the negativity in almost all patients with MCL (**Table 2**).

**Table 2:** CD200 expression and differential diagnosis in chronic B-cell leukemias: published data at a glance.

Reference	No. Patients	Samples evaluated	CLL	MCL	HCL	HCL-v	MZL	FL	LPL	Other B-cell neoplasms
Palumbo et al. (31)	91	PB	79/79 (100%)	0/14 (0%)	–	–	–	–	–	–
Brunetti et al. (30)	10	PB; BM	–	–	10/10 (100%)	–	–	–	–	–
Dorfman and Shahsafaei (32)	73	BM, LN^#^	21/21 (100%)	0/10 (0%)	12/12 (100%)	–	0/10 (0%)	0/16 (0%)	8/10 (80%)	MALT: 0/4 (0%); B lymphoblastic leukemia/lymphoma: 10/10 (100%); DLBCL: 0/12 (0%); mediastinal large B-cell lymphoma: 8/8 (100%); BL: 0/8 (0%); MM: 10/13 (77%); HL: 12/13 (92%); nodular lymphocyte predominant HL: 0/14 (0%)
Bhatnagar et al. (33)	100	PB; BM	78/78 (100%)	0/7 (0%)	–	–	–	–	–	–
El Desoukey et al. (34)	49	PB	31/31 (100%)	0/4 (0%)	2/2 (100%)	–	0/4 (0%)	0/8 (0%)	–	–
Cherian et al. (35)	66	Not reported	–	–	–	–	–	–	–	Not reported data as percentage expression of CD200 in 51 cases of CLL/SLL and 15 cases of MCL. Most cases studied can be separated using a cut off of 1.10 for the CD200 MFI ratio
Kern et al. (36)	100	PB, BM	58/59 (98,3%)	2/14 (14,3%)	–	–	–	–	–	CLL/PL cases: 26/27 (96.3%)
Alapat et al. (37)	107	PB; BM; LN; body fluids	19/19 (100%)	0/4 (0%)	–	–	–	–	3/7 (43%)	B-ALL: 19/20 (95%); T-ALL: 0/5 (0%); MM: 37/52 (71%)
Pillai et al. (38)	180	BM, PB, LN; body fluids and other tissues	–	–	23/23 (100%)	1/1 0(10%)	–	–	–	Not specified the number of positive/negative cases but only reported MFI (with range) for each disease category. CLL/SLL showed CD200 with MFI of 5,965 compared with MCL and FL which had MFIs of 397 and 521, respectively.
Dasgupta et al. (39)	56	Not reported	28/28 (100%)	2/10 (20 %)	3/3 (100%)	–	–	–	–	4/8 (50%) not otherwise specified
El-Sewefy et al. (40)	40	Not reported	30/30 (100%)	1/10 (10%)	–	–	–	–	–	
Sandes et al. (41)	159	PB; BM; LN; CSF	56/56 (100%)**	0/14 (0%)	13/13 (100%)	–	6/6 (100%)	8/11 (73%)	2/4 (50%)	PL: 2/7 (28%) SRPBL: 1/1 (100%) CD5-CD10- unclassifiable NHL: 21/31 (68%) CD5+ NHL*** 9/23 (39%)
Challagundla et al. (42)	364	PB; BM; FNA; LN	119/119 (100%)	58/61 (95%)*	7/7 (100%)	–	9/26 (35%)°	°°	3/3 (100%)°°	
Karban et al. (43)	200	PB	200/20 (100%)	4/46 (8,7%)	–	–	–	–	–	–
Sorigue et al. (44)	248	PB; BM; LN; CE	106/106 (100%)	–	–	–	–	–	–	MBL-CLL like: 106/106 (100%); DLBCL: 7/32 (22%) of which 4/11 (36%) ABC; 3/20 (15%) GCB; 0/1 (0%) cell of origin not determined; 1/4 (25%) BL.
Lesesve et al. (45)	124	PB; BM	57/69 (83%)	0/10 (0%)	2/4 (50%)	–	1/16 (6%)	0/7 (0%)	–	MBL-CLL like: 8(13(61%); MBL-non-CLL like 1/3(33%); PL: 0/2 (0%)
Fan et al. (46)	374	PB; BM	268/271 (99 %)	1/31 (35%)	3/3 (100%)	–	75% strong	–	–	PL: 5/7 (%) weakly, 2/7 (%) strong
Naseem et al. (47)	77	Not reported	54/54 (100%)	1/6 (16%)	5/5 (100%)	–	–	1/2 (50%)	–	Other CLPD did not express CD200
Rahman et al. (48)	3	Not reported	–	–	–	3/3 (100%)	–	–	–	–
Rahman et al. (49)	160	PB; BM; FNA, ascites fluid	98/98 (100%)	0/24 (0%)	6/6 (100%)	0/1 (0%)	3/6 (50%)	2/4 (50%)	2/4 (50%)	DLBCL: 3/5 (60%); BL: 0/1 (0%); PBL: 0/1 (0%); CD5-CD10- undefined lymphomas: 4/10 (40%)
Ting et al. (50)	97	PB; BM; LN; pleural fluid	56/56 (100%)^§^	0/6 (0%)	2/2 (100%)	–	–	–	–	40 pts of which 22 diagnosed with lymphomas with subtype (not reported in the paper), and 18 unclassified (of which 10 diagnosed with lymphoma without subtype, and 8 non-CLL MBL)
Arlindo et al. (51)	124	PB; BM	61.1 (41.4/89.2)	3.5 (2.1/4.1)	220.3 (163.1/297.5)	36.1 (22.1/50.1)	8.3 (4.5/13.2)	2.6 (1.8/11.4)		Atypical CLL: 113.7 (70.4/122.2)
Mason et al. (52)	79	PB;BM;LN	–	–	34/34 (100%)	0/3 (0%)	1/22 (5%)	–	13/20 (65%)	
Starostka et al. (53)	188	PB; BM; LN; pleural fluid	158/161 (98.3%)	7.9%	–	–	63.6%	–	–	
Miao et al. (54)	653	PB; BM	355/365 (97%)	12/41 (29%)	1/1 (100%)	2/2 (66.7%)	8/20 (40%)	13/17 (76.5%)	30/35 (85.7%)	MALToma: 4/4 (100%); CD5+ and CD5- unclassified B-cell chronic lymphoproliferative disorders: 119/153 (78%)
Poongodi et al. (55)	77	PB; BM	54/54 (100%)	1/6 (16,7%)	5/5 (100%)	–	2/2 (100%)	1/2 (50%)	–	DLBCL: 1/3 (33.3%); unclassifiable lymphoma: 2/3 (66.7%); SLL: 1/1 (100%)
Favre et al. (56)	96	PB	84/84 (100%)	1/30 (3%)	7/7 (100%)	–	13/14 (93%)	–	–	SRPBL: 6/15 (40%)
Falay et al. (57)	339	PB; BM	295/306^ç^ (95.8%)	2/33 (6.1% dimly)	–	–	–	–	–	
D’Arena et al. (58)	427	PB; BM	312/322 (97%)	4/21 (19%)	15/15 (100%)	–	27/53 (51%)	3/12 (25%)	0/4 (0%)	
Debord et al. (59)	135	PB, BM	–	3/63 (5%)	–	–	–	–	–	68/72 (94%) low grade B-cell lymphoma not otherwise specified
Mora et al. (60)	120	PB	64/64 (100%)	1/5 (20%)	–	–	13/19 (68.4%)	2/2 (100%)	–	MBL: 14/14 (100%); SLL: 3/3 (100%); other B-CLPD (not otherwise specified): 9/13 (69.2%)^
Myles et al. (61)	307	PB; BM; LNH, other body fluid or tissue	231/241 (96%)	62/66 (94%)	–	–	–	–	–	
Soong et al. (62)	189	PB; BM	121/121 (100%)	1/10 (1%)	–	–	–	–	–	54/68 of B-chronic lymphoid leukemias, including MCL cases
El-Neanaey et al. (63)	50	PB	30/30 (100%)	–	5/5 (100%)	–	–	–	–	14/25 (56%) B-NHL not subclassified

CLL, chronic lymphocytic leukemia; MCL, mantle cell lymphoma; HCL, hairy cell leukemia; HCL-v, hairy cell leukemia variant; MZL, marginal zone lymphoma; FL, follicular lymphoma; LP, lymphoplasmacytic lymphoma; PL, prolymphocytic leukemia; SRPBL, splenic diffuse red pulp small B-cell lymphoma; NHL, non-Hodgkin’s lymphoma; HL, Hodgkin lymphoma; SLL, small lymphocytic lymphoma; DLBL, diffuse large B-cell lymphoma; ABC, activated B cell-like; GCB, center B cell-like; MM, multiple myeloma; PBL, plasmablastic lymphoma; PB, peripheral blood; BM, bone marrow; LN, lymph node; CE, cavity exudate; FNA, fine needle aspiration; CSF, cerebrospinal fluid; IHC, immunohistochemistry, MFI, mean fluorescence intensity.

*Three cases of MCL moderate or bright CD200 expression (all three cases showed cyclin CD1 overexpression by IHC; two cases had t(11;14)(q12:q32) by FISH and conventional cytogenetics also; one case showed del(14q31q32) in three of 20 metaphases. Flow cytometry immunophenotyping in all three cases showed an atypical pattern of CD23 expression, positive on most lymphoma cells).

**11 cases were atypical-CLL (CD23 absent or dimly expressed in six cases and the following antigens expressed with moderate/strong intensity: FMC-7 in eight cases; CD79b in five cases, and smIg in four cases).

***CD5+ NHL (non-CLL, non-MCL).

#CD200 expression was studied by means of immunohistochemistry (HIC).

°mostly nodal or MALT types.

°°90 FL cases were studied and a spectrum of CD200 expression ranging from negative to moderate was found (not reported the number of positive cases); only three LPL cases were studied with moderate expression of CD200.

^§^56 CLL of which 13 CLL-like MBL; 7 patients with CLL were atypical. Both typical and atypical were found all CD200 positive; MFIand percentage of CD200 expression were found not different; no differences were found between typical and atypical CLL also.

^ç^No difference between typical and atypical CLL. Multivariate analysis for MCL and atypical CLL discrimination, it was demonstrated that the most determinant molecule was CD200 (p<0.0001, 95% CI).

*MFI >1,000 was used to represent positive staining while MFI ≤1,000 to represent negative staining for CD200.

^CD200, added to the canonical 5 markers, improved the diagnostic accuracy of Matutes score from 86.7% to 92.5% (p<.01).

Focusing on the assessment of fluorescence intensity of CD200, Lesesve et al used the ratio of mean fluorescence intensity (MFI) of CD160/CD200 on leukemic cells/controls (45). CD160 is a glycosylphosphatidylinositol-anchored cell surface molecule belonging to the immunoglobulin superfamily that was found to be expressed in patients with CLL (64, 65). Only 60% of patients showed surface positivity for CD160. Both markers were positive in 55% of CLL but only in 2% of other B-cell neoplasms, and absence of both markers occurred in 12% of CLL and in 86% of other B-cell neoplasms.

Taken together, these data showed that CD200 has a high sensitivity for CLL diagnosis, being expressed in most of the CLL cases. However, it is expressed also in other B-cell malignancies, including MZL, HCL, and even some cases with indolent, non-nodal entity MCL, thus showing low specificity.

Some authors tried to implement the diagnostic ability of monoclonal antibodies-based scores using CD200 (58, 66–68). Recently, Köhnke et al proposed the so-called “CLLflow score” including CD200 in the Matutes score and showing an improvement in the specificity for the diagnosis of CLL (66). Briefly, the score is calculated by adding the percentage of CD200+ and CD23+/CD5+ B cells and then subtracting the percentages of CD79b+ as well as FMC7+ B cells. A score >0 is consistent with the diagnosis of CLL while a score ≤ 0 with the diagnosis of a non-CLL disease entity (67–69). In our hands a simplified score system for the diagnosis of CLL, in which only 4 markers are used (CD5, CD23, CD200, and SmIg), showed a higher sensitivity and specificity with respect to the Matutes score (58).

**Figure 3:**
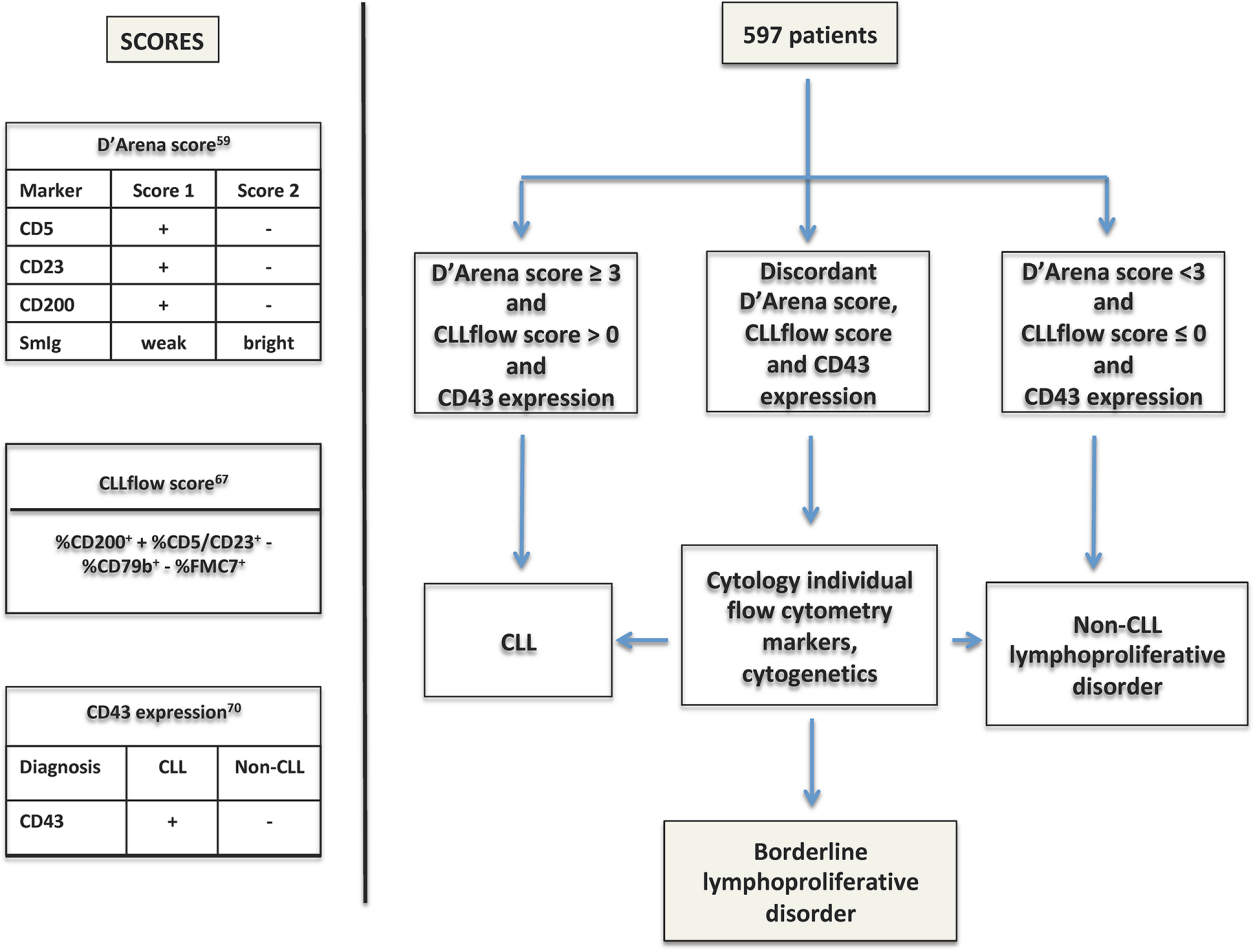
Overview of the study by Sorigue et al. (70). The algorithm of the study using the three diagnostic scores to assign a diagnostic category is explained in the text (58, 66, 69).

Interestingly, very recently Sorigue et al, using three immunophenotype-based diagnostic approaches (Matutes score, D’Arena score and CD43 expression) analyzed 597 patients with a chronic lymphoproliferative disorder (CLPD) and found that patients with concurring CLL-like or non-CLL like results according to the three diagnostic strategies were diagnosed with CLL (n = 441) and non-CLL (n =99), respectively (**Figure 3**) (69, 70). ‘Discordant’ patients (n = 57) were further re-evaluated taking into account individual cytometric markers and cytogenetics data and only 16 patients (2.7%) were not assigned to a reasonable diagnosis (**Figure 3**). The latter cases were considered “borderline lymphoproliferative disorders”, a loosely-defined concept that would include any chronic lymphoid disorder in which the diagnosis of CLL cannot be either made or ruled out.

In another recent study, Sorigue et al conducted a systematic review of the use of CD200 in the differential diagnosis of CLPDs (71). They evaluated the positive predictive value of CD200 on the prevalence of the disorders evaluated for the differential diagnosis. Twenty-seven publications were included in this systematic review (accounting for 5,764 patients). The median positivity rate and percentage of CD200 positive cells in patients with CLL was 100% and 95%, respectively, whereas it was 4% and 8% in MCL, and 56% and 62% in other CLPDs. The authors concluded that CD200 is a suboptimal marker in discriminating CLL from CLPDs other than MCL. These findings suggest that assessment of CD200 within scores rather than as a single marker, as reported by some authors, would be more useful (66–68).

In addition, some authors investigated whether a different expression of CD200 between CLL and monoclonal B-cell lymphocytosis (MBL) exists. Sorigue et al demonstrated that all cases of both CLL and CLL-like MBL were CD200 positive. However, MBL cases showed a lower CD200 MFI than CLL. Moreover, both CLL with trisomy 12 and CLL-like MBL displayed lower CD200 MFI than CLL with other cytogenetic abnormalities. In contrast, Rawstron et al did not find any difference in expression of a large body of markers, including CD200, between CLL-type MBL and CLL (72). However, differences in the expression of other markers were observed, including lower expression of CD38, CD62L, and CD49d and higher expression of LAIR-1, CXCR5, and CCR6 on CLL-type MBL compared to CLL. In conclusion, despite CLL-type MBL being phenotypically identical to CLL for a large body of antigens, certain differences exist, particularly with respect to proteins involved in the homing to lymphoid tissue.

## CD200 and Prognosis in CLL

Little is known about the prognostic significance of CD200 expression in CLL. As a matter of fact, conflicting results have been reported so far. Few data have been published and both percentage of CD200 positivity and fluorescence intensity have been used to estimate CD200 expression on CLL B-lymphocytes. Firstly, Wang et al in 2014 identified two distinct groups of CLL patients according to the expression of CD200 on bone marrow B cells (CD200 low group: <50%; CD200 high group: ≥ 50%) (73). As reported in **Table 3**, correlations were found with some clinical-biological features of CLL patients with <50% CD200+ cells. In contrast, using the same methodological approach, El Din Fouad et al found other correlations in patients with a lower percentage of CD200+ CLL cells (<50%) (74). Moreover, while the former study did not report data on TTT, response to therapy or OS, the latter found no correlation with response to treatment or OS. In a large cohort of patients with CLL, Miao et al, evaluating the CD200 mean fluorescence intensity (MFI) instead of the percentage of positivity, found that patients with lower CD200 MFI had a significantly shorter TTT with respect to patients with higher CD200 MFI (75). However, no correlation was found between CD200 MFI and OS, and CD200 MFI did not maintain its predictive value on TTT in multivariate analysis. More recently, using a more standardized flow cytometric approach, we measured the CD200 MFI on CD19+ and CD19- lymphocyte subpopulations, calculating the relative fluorescence intensity (RFI) as a ratio of the CD200 MFI on CD19+ and CD19- cells (76). Lower and higher CD200 RFI values were found to be associated with del11q and del13q14, respectively. In addition, CD200 RFI greater than the mean cohort value was detected in patients with longer OS. However, these data have to be confirmed in patients with a longer follow-up.

**Table 3:** CD200 expression and prognosis in CLL.

Reference	No. Patients evaluated	CD200 positivity cut-off	Correlations
Wang et al. (73)	40	50% of B-cells (<50%: CD200 low group; ≥50% CD200 high group)	<50% of CD200-positive B-cells positively correlated with younger age, female gender, lower WBC, lower lymphocyte absolute and percentage number, lower lymph node involvement, lower ZAP-70 positive cells, early disease stage. No data on TTT, response to therapy and OS.
El Din Fouad et al. (74)	43	50% of B-cells	>50% of CD200-positive B-cells positively correlated with older age, lymphocytosis, hepatomegaly, splenomegaly, higher Rai and Binet stage. No correlation with response to treatment and OS.
Miao et al. (75)	307	CD200 MFI cut-off: 189.5 (<lower group; ≥higher group)	Lower CD200 MFI positively correlated with shorter TTT.
D’Arena et al. (76)	105	CD200 RFI cut-off: 13	Lower CD200 RFI positively correlated with del11q and negatively correlated with del13q14. CD200 RFI greater than the mean value of the entire cohort positively correlated with longer OS.

WBC, white blood cell count; TTT, time to treatment; OS, overall survival; MFI, mean fluorescence intensity; RFI, relative fluorescence intensity (ratio of MFI of CD200 on CD19+ lymphocytes/MFI of CD200 on CD19- lymphocytes).

In summary, conflicting results have been reported on the prognostic role of CD200 expression in CLL so far. Methodological and sampling differences, such as the analysis of percentage of positive cells versus MFI, bone marrow versus peripheral blood cells, and different flow cytometry instrument settings and/or gating strategy, could account for these differences. Further studies are needed to better understand this issue before reaching definitive conclusions.

## Conclusions and Future Directions

CD200 expression is useful in better classifying B-cell CLPDs. The addition of CD200 to flow cytometry marker panels addressing the diagnosis of this heterogenous group of B cell neoplasms may be particularly helpful in distinguishing some disease entities, in particular CLL and MCL, whose clinical behavior and prognosis are quite different. On the contrary, expression of CD200 does not appear to have a relevant role as a prognostic indicator in CLL according to data published so far, despite limited in number. However, this issue needs to be better addressed by studies on a larger cohort of patients and using standardized methodologies. Finally, the potential role of CD200 as a therapeutic target must be taken into account in particular for diseases that highly express CD200, such as CLL and HCL (24, 77). In fact, CD200 is known to act as an immunosuppressive molecule that is upregulated on primary CLL B-cells (18 *).* Moreover, an elegant work by Kretz-Rommel and co-workers demonstrated that CD200 expression by tumor cells suppresses antitumor responses in an animal model (22). In a similar fashion, Gorczynski et al showed that by manipulating CD200:CD200R interactions it is possible to cure local tumors and distant metastases in a murine breast cancer model (78, 79). Very recently, the first-in-human study investigating the therapeutic use of the recombinant humanized monoclonal anti-CD200 antibody samalizumab in 23 patients with advanced CLL and 3 patients with MM was published (phase 1 study NCT00648738) (80). Treatment was associated with mild to moderate adverse events and resulted in a dose-dependent decrease in CD200 expression on CLL cells. Decreased tumor burden was also observed in 14 CLL patients, with one of them achieving a durable partial response, while 16 patients maintained a stable disease. These results, although preliminary, suggest that samalizumab could represent an immune checkpoint inhibitor with activity in CLL.

Finally, there is some evidence that serum levels of soluble CD200 may be related to disease progression and prognosis in patients with CLL (79, 81, 82 *).* In particular, Wong et al showed that CD200 can be released from CD200+ neoplastic cells by ectodomain shedding (81). This event is regulated by ADAM28 (79, 82 *).* Interestingly, both the membrane and the soluble form of CD200 is able to engage CD200R, which in turn can result in increased tumor growth (20, 83 *).* This happens by means of reduction of immune reactivity and/or increment of induction/activation of regulatory T cells in the tumor microenvironment (83, 84 *).* Taken together, more and more convincing data have been reported in the literature from which the relevant role of CD200 is emerging not only as a diagnostic and prognostic tool but also as a potential therapeutic target in various neoplasms including CLL.

## Author Contributions

GD’A, GP, VD, FA, SD, DE, AS, and LL designed the project, revised the scientific literature, and wrote the paper. GD’A, GP, ES, GM, and OV followed patients with chronic lymphocytic leukemia. FL, FD’A, TS, and LV performed flow cytometric studies. All authors contributed to the article and approved the submitted version.

## Conflict of Interest

The authors declare that the research was conducted in the absence of any commercial or financial relationships that could be construed as a potential conflict of interest.
